# Editorial for the Special Issue “New Drugs for Breast Cancer Treatment”

**DOI:** 10.3390/ijms231810265

**Published:** 2022-09-06

**Authors:** Filippo Acconcia

**Affiliations:** Department of Sciences, University Roma Tre, Viale Guglielmo Marconi, 446, I-00146 Rome, Italy; filippo.acconcia@uniroma3.it; Tel.: +39-0657336320; Fax: +39-0657336321

Breast cancer (BC) is the deadliest neoplastic disease for women worldwide. Progress in the classification of this neoplasia has firmly demonstrated its heterogeneous nature. According to different classification methods, a plethora of individual types of BCs exist and must be approached in different ways. Thus, perhaps, BC represents one of the best examples of using a patient- and tumor-driven personalized approach for treatment [1].

In this respect, different assets exist and are used in the clinic: for estrogen receptor α (ERα)-positive BC, endocrine therapy (ET) is implemented and has proven efficacious in increasing patient survival. However, it is well known that a significant fraction of women treated with ET drugs (e.g., 4OH-tamoxifen) relapse into a metastatic disease (that is heterogenous itself in nature), which is by and large incurable. On the other hand, a very serious clinical problem is the management of ERα-negative tumors, for which very limited pharmacological options are available. Unfortunately, patients with this kind of disease display a poor prognosis and a short life expectancy [1].

For this reason, it is imperative to identify new drugs for breast cancer treatment, especially from the perspective of the identification of tumor-driven and patient-specific drugs. Therefore, this Special Issue aims to provide experimental and theoretical clues for the rational identification of novel strategies for BC treatment.

A total of 14 papers are included in this Special Issue, with 10 of them being research articles reporting new compounds potentially usable for novel treatments of ERα-positive and ERα-negative BCs. In particular, the readers of this Special Issue will learn about the anti-tumor activity of neosynthesized, natural, and already-available drugs that could be used as new anti-BC drugs. Indeed, the contributing authors have reported the antiproliferative activity of berberine and troglitazone derivatives, resveratrol analogs, atoquavone, clotrimazole, fenticonazole, compounds specifically targeting mitochondrial activity, and valproic acid, as well as NAMPT and carbonic anhydrase IX inhibitors [2,3,4,5,6,7,8,9,10,11].

Four additional articles in this Special Issue are reviews. In two of them, the contributing authors discuss the intriguing possibility of beginning tumor treatment during the process of diagnosis [12], or even during the surgical resection procedure [13]. In the last two contributions to this Special Issue, the researchers review all the up-to-date options for BC management according to the molecular classification of breast tumors [14] and propose alternative pharmacological targets for BC treatment [15].

Given the information provided in these works, it is evident that BC sufferers can expect new hopes for the innovative treatment of this disease. Although a great amount of work remains to be completed, research efforts will certainly grant novel personalized treatment for this neoplasm.

Finally, the Guest Editor would like to thank all authors for their excellent contributions and the Editorial Board for their support.

## References

[1] Tsang J.Y.S., Tse G.M. (2020). Molecular Classification of Breast Cancer. Adv. Anat. Pathol..

[2] Geoffroy M., Lemesle M., Kleinclauss A., Mazerbourg S., Batista L., Barberi-Heyob M., Bastogne T., Boireau W., Rouleau A., Dupommier D. (2022). AB186 Inhibits Migration of Triple-Negative Breast Cancer Cells and Interacts with alpha-Tubulin. Int. J. Mol. Sci..

[3] Estrada-Perez A.R., Rosales-Hernandez M.C., Garcia-Vazquez J.B., Bakalara N., Fromager B., Correa-Basurto J. (2022). Untargeted LC-MS/MS Metabolomics Study on the MCF-7 Cell Line in the Presence of Valproic Acid. Int. J. Mol. Sci..

[4] Janoniene A., Mazutis L., Matulis D., Petrikaite V. (2021). Inhibition of Carbonic Anhydrase IX Suppresses Breast Cancer Cell Motility at the Single-Cell Level. Int. J. Mol. Sci..

[5] Andreidesz K., Szabo A., Kovacs D., Koszegi B., Bagone Vantus V., Vamos E., Isbera M., Kalai T., Bognar Z., Kovacs K. (2021). Cytostatic Effect of a Novel Mitochondria-Targeted Pyrroline Nitroxide in Human Breast Cancer Lines. Int. J. Mol. Sci..

[6] Gupta N., Gaikwad S., Kaushik I., Wright S.E., Markiewski M.M., Srivastava S.K. (2021). Atovaquone Suppresses Triple-Negative Breast Tumor Growth by Reducing Immune-Suppressive Cells. Int. J. Mol. Sci..

[7] Xulu K.R., Augustine T.N. (2021). Antiplatelet Therapy Combined with Anastrozole Induces Features of Partial EMT in Breast Cancer Cells and Fails to Mitigate Breast-Cancer Induced Hypercoagulation. Int. J. Mol. Sci..

[8] Cipolletti M., Bartoloni S., Busonero C., Parente M., Leone S., Acconcia F. (2021). A new anti-estrogen discovery platform identifies FDA-approved imidazole anti-fungal drugs as bioactive compounds against ERα expressing breast cancer cells. Int. J. Mol. Sci..

[9] Iacopetta D., Lappano R., Mariconda A., Ceramella J., Sinicropi M.S., Saturnino C., Talia M., Cirillo F., Martinelli F., Puoci F. (2020). Newly Synthesized Imino-Derivatives Analogues of Resveratrol Exert Inhibitory Effects in Breast Tumor Cells. Int. J. Mol. Sci..

[10] Pierpaoli E., Piacenza F., Fiorillo G., Lombardi P., Orlando F., Salvatore C., Geroni C., Provinciali M. (2021). Antimetastatic and Antitumor Activities of Orally Administered NAX014 Compound in a Murine Model of HER2-Positive Breast Cancer. Int. J. Mol. Sci..

[11] Podsednik A., Jiang J., Jacob A., Li L.Z., Xu H.N. (2021). Optical Redox Imaging of Treatment Responses to Nampt Inhibition and Combination Therapy in Triple-Negative Breast Cancer Cells. Int. J. Mol. Sci..

[12] Vahidfar N., Aghanejad A., Ahmadzadehfar H., Farzanehfar S., Eppard E. (2021). Theranostic Advances in Breast Cancer in Nuclear Medicine. Int. J. Mol. Sci..

[13] Raigon Ponferrada A., Guerrero Orriach J.L., Molina Ruiz J.C., Romero Molina S., Gomez Luque A., Cruz Manas J. (2021). Breast Cancer and Anaesthesia: Genetic Influence. Int. J. Mol. Sci..

[14] Lau K.H., Tan A.M., Shi Y. (2022). New and Emerging Targeted Therapies for Advanced Breast Cancer. Int. J. Mol. Sci..

[15] Noureddine L.M., Tredan O., Hussein N., Badran B., Le Romancer M., Poulard C. (2021). Glucocorticoid Receptor: A Multifaceted Actor in Breast Cancer. Int. J. Mol. Sci..

